# Genome Sequence of Bellavista Virus, a Novel Orthobunyavirus Isolated from a Pool of Mosquitoes Captured near Iquitos, Peru

**DOI:** 10.1128/genomeA.01262-16

**Published:** 2016-11-10

**Authors:** Jun Hang, Yu Yang, Robert A. Kuschner, Julio Evangelista, Helvio Astete, Eric S. Halsey, Tadeusz J. Kochel, Brett M. Forshey

**Affiliations:** a Walter Reed Army Institute of Research, Silver Spring, Maryland, USA; b U.S. Naval Medical Research Unit No. 6, Iquitos and Lima, Peru

## Abstract

A novel orthobunyavirus, Bellavista virus, was isolated from *Culex (Melanoconion)* portesi mosquitoes in the Bellavista neighborhood of Iquitos, Peru, in 2009. The assembled segment L, M, and S sequences of strain PRD0552 are 6,950, 4,469, and 1,256 bases in length, respectively, comprising complete protein-coding sequences and partial terminal untranslated sequences.

## GENOME ANNOUNCEMENT

Many arthropod-borne viruses (arboviruses) are human pathogens (1, 2). Group C arboviruses were first isolated in acute febrile illness (AFI) patients in the 1950s and subsequently classified as members of genus *Orthobunyavirus* (family *Bunyaviridae*) (3). Orthobunyaviruses have been isolated from humans, other mammals, and arthropods in many regions worldwide (4–8). An epidemiological study in western South America identified AFI patients with orthobunyavirus infection in and around the city of Iquitos, located in the Amazon basin of northeastern Peru (9). To identify potential vectors of orthobunyaviruses in the region, we collected mosquitoes using CO_2_-baited light traps in four sites in two neighborhoods, Bellavista-Nanay and San Juan, in peri-urban areas of Iquitos (10).

Mosquitoes were pooled by species, location, and date; triturated; and inoculated onto mosquito (*Aedes albopictus* C6/36) and mammalian (African green monkey Vero 76) cell cultures (10). In one pool of *Culex (Melanoconion)* portesi collected in the Bellavista-Nanay neighborhood in November 2009 (pool PRD0552), orthobunyavirus infection was indicated by cytopathic effect and indirect immunofluorescent assay in inoculated Vero 76 cells using polyclonal antibodies against group C orthobunyaviruses. Nucleic acids of isolate PRD0552 were extracted from the supernatant of Vero 76 cell culture and subjected to random reverse transcription, random PCR amplification and high-throughput pyrosequencing using GS FLX system (Roche 454 Life Sciences, Branford, CT) (11, 12). Sequence data were assembled and analyzed using Roche GS analysis software v2.9 (Roche 454 Life Sciences), BLAST programs (http://blast.ncbi.nlm.nih.gov/Blast.cgi), and software Geneious 8.1.7 (Biomatters, Auckland, New Zealand).

The genome sequence for PRD0552 was assembled using 34,931 reads, in total 8,389,524 bases, with average sequence alignment depth of 660-fold. The assembled large (L) segment sequence of the genome has 6,950 bases with a complete open reading frame (ORF) encoding a 2,248 amino acids protein. The L protein has amino acid identity of 67.3% with RNA-dependent RNA polymerase (RdRp) of Enseada virus (AMR98952), a mosquito orthobunyavirus identified in Brazil (13) and 66.5% with RdRp of Caraparu virus (AGW82149), a human orthobunyavirus pathogen identified in Peru (14). The medium (M) segment sequence has 4,469 bases encoding one complete ORF for a 1,436 amino acids polyprotein, consisting of nonstructural protein NSm and glycoproteins Gn and Gc. The polyprotein is homologous with M proteins of human and mosquito orthobunyaviruses, including Guama virus (AKO90175) and Catu virus (ALS54743) (15), albeit with amino acid identity of 62% or lower. The small (S) segment sequence has 1,256 bases encoding two complete ORFs for a putative 236 amino acid nucleocapsid protein and a putative 95 amino acid nonstructural protein NSs. These two proteins had low similarity with known orthobunyaviruses, including 51.0% and 41.5% amino acid identities with the respective homologs (AMR98955 and AMR98954) in Enseada virus (14). The low identities with known orthobunyaviruses for the encoded gene products indicate that the isolate represents a novel virus, which we provisionally term Bellavista virus, after the neighborhood where the mosquitoes were collected.

### Accession number(s).

The segment L, M, and S sequences for the Bellavista virus genome were deposited in GenBank under accession numbers KX161718 to KX161720.
